# Nanophotonic detection of freely interacting molecules on a single influenza virus

**DOI:** 10.1038/srep12087

**Published:** 2015-07-10

**Authors:** Pilgyu Kang, Perry Schein, Xavier Serey, Dakota O’Dell, David Erickson

**Affiliations:** 1 Sibley School of Mechanical and Aerospace Engineering, Cornell University, Ithaca, New York 14853, USA; 2 School of Applied and Engineering Physics, Cornell University, Ithaca, New York 14853, USA

## Abstract

Biomolecular interactions, such as antibody-antigen binding, are fundamental to many biological processes. At present, most techniques for analyzing these interactions require immobilizing one or both of the interacting molecules on an assay plate or a sensor surface. This is convenient experimentally but can constrain the natural binding affinity and capacity of the molecules, resulting in data that can deviate from the natural free-solution behavior. Here we demonstrate a label-free method for analyzing free-solution interactions between a single influenza virus and specific antibodies at the single particle level using near-field optical trapping and light-scattering techniques. We determine the number of specific antibodies binding to an optically trapped influenza virus by analyzing the change of the Brownian fluctuations of the virus. We develop an analytical model that determines the increased size of the virus resulting from antibodies binding to the virus membrane with uncertainty of ±1–2 nm. We present stoichiometric results of 26 ± 4 (6.8 ± 1.1 attogram) anti-influenza antibodies binding to an H1N1 influenza virus. Our technique can be applied to a wide range of molecular interactions because the nanophotonic tweezer can handle molecules from tens to thousands of nanometers in diameter.

Investigating interactions between a virus and its antibody plays a key role in pathogen control and prevention^1^,^2^,^3^. It allows identification of the pathogen type and determination of the virulence. A number of optical^4^,^5^,^6^, electrochemical^7^,^8^, and mechanical^9^ biosensor-type techniques have been developed for this purpose. In addition to detection, recently developed imaging-based techniques^10^,^11^,^12^ are capable of giving quantitative information such as the size and mass of single viruses. These methods enable label-free detection, but they rely on immobilizing a specific antibody on a sensing area^13^,^14^,^15^ and thus constrain the active binding to the target. Especially in multivalent bindings, this restriction prevents an accurate measurement of their affinity and binding capacity. As a result, the measured binding kinetics may not be representative of what occurs in free solution^16^,^17^. Instruments developed using techniques such as nanoparticle tracking analysis (NTA) and dynamic light scattering (DLS), afford free solution conditions and in principle can measure particle size, but the techniques have major drawbacks in the reproducibility of size distributions and peak resolution of mean particle sizes^18^. Furthermore, these techniques are not sensitive enough to detect a difference in size of only a few nanometers such as in the case of measuring a particle before and after the binding of a monolayer of antibodies. Biomolecular particles vary in size and shape, and the single particle analysis may provide more accurate measures especially for particles with largely different sizes like virus and antibodies. Most recently, nanoaperture optical tweezers based on plasmonic trapping using nanoholes in metal films have been emerging in label-free and free-solution approaches for investigating biomolecular interactions including real-time dynamics and binding kinetics at the single molecule level^19^,^20^,^21^,^22^. The nanoaperture optical trap approach is useful but has attempted to probe the interactions between relatively small proteins of tens of kDa (*R*_h_ < 5 nm) with nanoholes with a diameter of hundreds of nanometers that is predetermined in fabrication.

In this paper we present a method that detects unrestricted interactions between biomolecules. Using the near-field optical trap we have developed^23^,^24^, this technique provides quantitative analysis of the interactions at the attogram scale. Above all, our technique is based on a single particle analysis with small uncertainty in a measurement, not requiring statistical results over a large number of virus particles^5^. Briefly, this method exploits the fact that the optical force exerted on a trapped particle is proportional to the particle’s volume and polarizability. The trap stiffness (the effective spring constant for the restoring optical force) can be extracted from the Brownian fluctuations of the trapped particle^25^. Thus, by observing these fluctuations, we can detect the binding of a partner biomolecule to the trapped particle (Fig. 1). This enables measurement of the binding interactions without restricting them by immobilizing or labeling either of the interacting biomolecules. In addition, the model that we have developed for the effective polarizability of the binding complex enables accurate measurements of the affinity and stoichiometry of the interactions (Fig. 2a – i,ii). In this paper, we first demonstrate the method using commercially available protein-coated fluorescent polymer nanoparticles that allow monitoring of the Brownian fluctuations. We perform an antibody binding assay in which one interacting antibody is coupled to the surface of a nanoparticle and the partner antibody freely moving in solution binds to it. This assay shows that our detection method is applicable to a biological assay between unlabeled interacting biomolecules by means of a fluorescent probe particle. Next, we demonstrate the detection of the binding interactions of antibodies to a single human influenza A virus, measuring stoichiometry of the specific antibody. Most importantly, we carry out the virus assay by utilizing a near-field light scattering technique^26^. The technique enables observation of Brownian fluctuations of the virus within an optical trap without either fluorescent labeling to the virus particle or conjugation to a fluorescent emitter.

We describe the result of molecular binding to the target using an effective sphere model of antibody-particle complexes (Fig. 2a – i,ii). This model is developed for particles in the Rayleigh regime, where the particle is small relative to the wavelength of light (the particle diameter 2*r* ≤ λ/π *n*_*m*_)^27^,^28^,^29^. The effective polarizability of the sphere allows us to describe the interactions with the known applied optical force from the equation^24^,^25^. To accurately measure affinities and stoichiometries of the interactions, we obtain the relationship between the relative trap stiffness and the radius increase as following. First, we relate the optical force exerted on a non-absorbing (*ε* ≈ *n*^2^) Rayleigh particle to the radius of the particle by the effective polarizability of the particle *α*_*eff*_ as in *F*_*trap*_ *=* 2π∇*I*_*o*_*α*_*eff*_ /*c*, where *c* and λ are the speed and wavelength of light, and *I*_*o*_ is the incident intensity. Noting that binding of biomolecules to the trapped particle changes the polarizability, we describe the change by using the core-shell model of a coated sphere to account for the effective polarizability of dissimilar dielectric constituent materials, for example, antibodies and polymer (Fig. 2a – i), and antibodies and virus (Fig. 2a–ii) in our assays, expressed as


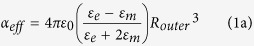



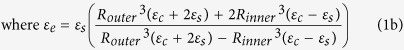


is the effective dielectric constant of the sphere, ε_*c*_, ε_*s*_, and ε_*m*_ are the dielectric constants of the core, shell, and medium respectively, *R*_*outer*_ is the core-shell radius, and *R*_*inner*_ is the core radius^30^. To evaluate ε_e_ ( = *n*_e_^2^) in equation (1b), the following values for the refractive indices are used: *n*_*PS*_ = 1.59 for the core of a polystyrene (PS) particle, *n*_*IgG*_ = 1.41 for the shell of an antibody layer^15^,^31^, and *n*_*virus*_ = 1.48 for the core of an influenza virus^12^. To estimate the thickness of the binding layer using the analytical model, the refractive index of an IgG layer was used from the literature^15^,^31^ where they measured the value experimentally. The adsorbed antibody layer is mainly composed of water as illustrated in Figure S4 and calculated for the fractional occupancy of an antibody layer. Thus the value of 1.41 we used is reasonable in that it is close to the refractive index of water, 1.33. Thus we relate the change in the polarizability to the change in size using equation (1a).

In our experiments, we optically trap a particle using a photonic crystal (PhC) resonator (Fig. 2b – i,ii, and Supplementary Information). We use the equipartition method^32^ to extract the trap stiffness from the positional variance of the particle within the optical trap using video tracking analysis, using the relation *k*_*trap*_ = 2 *k*_*B*_*T*/*r*_*rms*_^2^, where *k*_*B*_ is the Boltzmann constant, *T* is the temperature in K, and *r*_*rms*_^2^ = (1/*n*)∑(*x*^2^ + *y*^2^) is the variance of *n* instantaneous positions^27^. Assuming small displacements within the optical trap that give *k*_*trap*_ = ∂*F*_*trap*_/∂*r*, we obtain the equation that relates the trap stiffness to the effective polarizability *k*_*trap*_ = 2*πα*_*eff*_ (∂^2^*I*_*o*_/∂*r*^2^), substituting this into the relative trap stiffness *f* = (*k*_*trap,*ΔR_/*P*_ΔR_)/(*k*_*trap,*0_/*P*_0_), that yields *α*_*eff,*Δ*R*_/*α*_*eff,*0_. To summarize, we can relate the ratio of the power normalized trap stiffnesses to the ratio of the effective particle polarizabilities before and after binding as the equation (2) below:


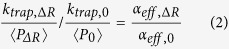


where *P* is the power, *k*_trap_ is the trap stiffness, subscript 0 denotes an initial measurement, subscript ∆*R* denotes the measurement at saturation, and the < > brackets indicate time averages over a measurement window.

Finally, we have obtained a transcendental equation (3) that relates the measured power normalized trap stiffness to the change in radius of the particle, allowing us to determine the thickness of the bound antibody layer. Therefore we determine the radius increase ∆*R* with the following equation:


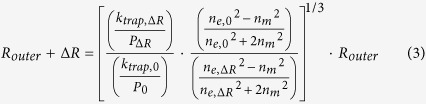


where *P* is the laser power and all other variables are as previously defined. In the Supplementary Figure S3, the change of the relative stiffness *f* as a function of varying initial size of a particle is plotted with respect to two different sizes of antibody, IgG and IgM (*d*_*IgG*_ = 5.8 nm and *d*_*IgM*_ = 10.6 nm), binding to an antibody-coated particle as well as an influenza virus.

Note that the analytical model for determining the thickness of the bound layer is derived for the Rayleigh regime where the particle diameter 2*r* ≤ λ/π*n*_*m*_ ≅ 254 nm, where *n*_*m*_ is refractive index of medium, *r* is radius of a particle, and λ is wavelength of light. Ashkin *et al*.^27^ experimentally demonstrated the validity of the condition by optically trapping varying size of polymer and silica particles from 10 μm down to ~25 nm that range from the Rayleigh regime to the full Mie regime, through the transition region. To be more specific, Ashkin *et al*. showed that the condition applies not only in the Rayleigh regime, but also in the moderate transition regime that is closely Rayleigh. In this regard, the virus particles we investigated are well within the Rayleigh regime in that the virus diameter 2*r* ≈ 100 nm < 254 nm. Although a 270 nm IgG coated fluorescent particle is not strictly Rayleigh by a factor of ~1.06 in the Rayleigh criteria, it is close enough to apply our model in consideration of a diameter variation (see Methods for particle information).

## Results and Discussion

**Figure 2:**
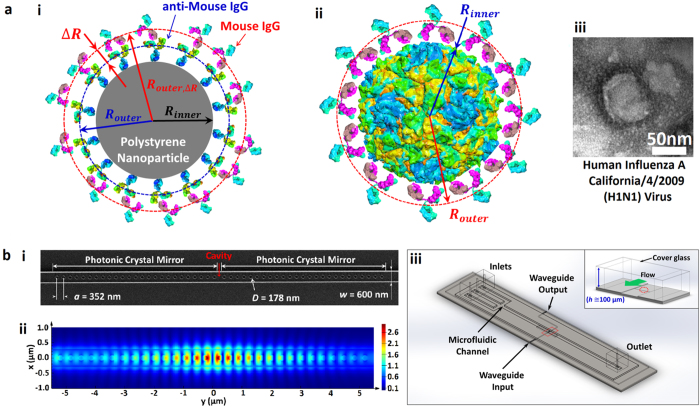
Effective sphere model of antibody-particle complexes and the nanophotonic tweezer (**a**) (i) A core-shell model of a goat anti-mouse IgG-coated polystyrene particle and bound mouse IgGs to the anti-mouse IgGs. (ii) A core-shell model of a virus and bound antibodies. Viral envelope is not shown for simplicity. (iii) A TEM image of an influenza virus (see SI). (**b**) (i) SEM image of the photonic crystal resonator. (ii) 3D FDTD simulation illustrating the strong field confinement within the resonator cavity. (iii) 3D schematics of an integrated optofluidic device. The inset shows a cross sectional view noted with a red box.

**Figure 3:**
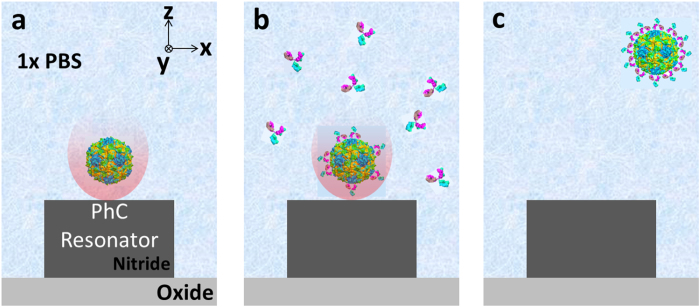
Schematics of procedure of the bioaffinity assay. The procedure involves three primary step from (**a**–**c**). (**a**) An influenza virus particle is optically trapped by a photonic crystal (PhC) resonator. Once the virus is trapped, an initial measurement of the trap stiffness is conducted. (**b**) The flow in a microchannel is switched from virus solution to antibody dispersed solution. Antibodies in the following solution bind to the trapped virus. The binding is saturated for 30 min in a stopped flow after the flow switching. (**c**) The second measurement of the trap stiffness after the binding is carried out. After the measurement, the antibody-virus complex is released when the laser is turned off.

In our experiments, we determine the power-normalized relative trap stiffness for equilibrium binding affinity after an incubation period of approximately 30 minutes. In general, the interactions occur in a short time, but the relatively long incubation time in our experiments ensures saturation of binding even at relatively low concentrations of antibody solutions, yielding a saturated layer after 30 minutes of adsorption^31^ (see the Methods for preparations). During this time, the solution of binding antibody is flowed over a trapped particle using a microfluidic channel (Fig. 2b – iii and Fig. 3, see Supplementary Information for details about preparation of the microchannel).

**Figure 4:**
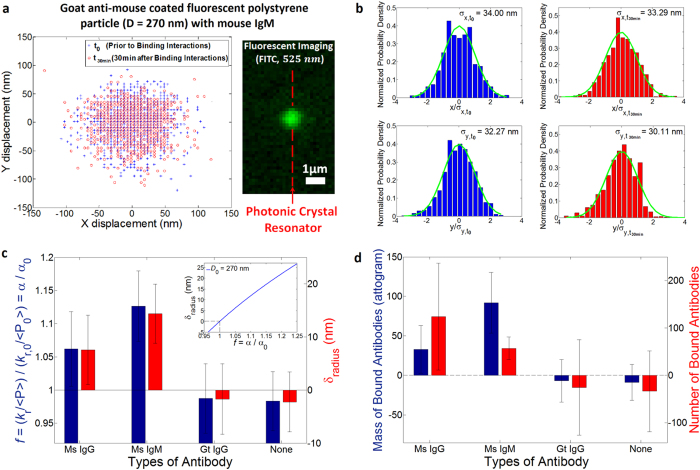
Binding of antibodies to goat anti-mouse IgG coated on the surface of fluorescent polystyrene particle (*D*_*mean*_ = 277.3 nm, *σ* = 36.2 nm) (**a**) Tracking trajectories within the optical trap before (blue crosses) and after (red circles) the binding with mouse IgM. The image on the right side captured by a CCD camera with a FITC cube shows a IgG-coated colloid trapped at the resonator cavity. (**b**) Normalized probability density histograms of *x* (top) and *y* (bottom) displacements before (left, blue bars, *k*_*r,t*0_ = 3.683 × 10^−3^ *pN/nm*, and *k*_*r,t*0_/ < *P*_0_ > = 1.723 *pN/nm∙W*, ε = 1.44) and after (right, red bars, *k*_*r,t*1_/ < *P*_1_ > = 1.851 *pN/nm∙W*, ε = 1.667 where the eccentricity of the optical trap^40^ is defined as ε = [*k*_*y*_^2^ − *k*_*x*_^2^/*k*_*y*_^2^]^1/2^ when *k*_*y*_ > *k*_*x*_ to quantify trapping force uniformit_*y*_) binding with mouse IgM. Green curves are Gaussian fits to the histograms. Note that < *P*_TE_ > is normalizing power to account for a TE mode of field coupled in the resonator cavity that involves in a radial optical trap in x-y plane. (**c**) Measured relative power-normalized trap stiffness and radius increases for different solutions of mouse IgG (*N* = 3), mouse IgM (*N* = 4), and goat IgG (*N* = 3), and a buffer (*N* = 5). *N* represents a number of independently performed experiments. The inset exhibits an analytical plot of the radius change to the relative polarizability for a IgG-coated colloid with *D*_0_ = 270 nm. (**d**) Stoichiometries of the antibodies to the colloid. All error bars are determined by (∑*σ*_*f*_^2^)^1/2^/*N* (see SI for details on *σ*_*f*_, standard deviation of an independent experiment).

To demonstrate the detection method, we first measure specific binding between a fluorescent polystyrene bead coated with goat anti mouse IgG and antibodies in solution. We compare our measured binding capacity of antibodies with the manufacturer’s quoted value (Spherotech Inc.). In our experiment, we first measure the Brownian fluctuations of the protein-coated bead in a saline solution with no antibody present (Fig. 3a). Then, we flow specific antibodies into the channel and wait 30 minutes with no imposed fluid flow for the binding to saturate (Fig. 3b). Following saturated binding, the measured change in radius corresponding to the bound layer thickness and the known binding capacity of the beads allow us to determine the number and mass of bound biomolecules in the assay. Note that this layer contains both antibodies and bound water molecules and therefore the measured number of antibodies accounts for this as the finite antibody size^31^. (See Figure S4 and find more details about the particles and determination of the density in Methods and SI respectively). The position fluctuations are measured using fluorescence microscopy (Fig. 4a). Typical measurements are shown in Fig. 4b.

Changes in power-normalized trap stiffness and radius increases of an IgG coated colloid are compared for solutions of mouse IgG, mouse IgM, goat anti-rabbit IgG, and a buffer (Fig. 4c). We use our model to compute the radius increase from the power-normalized relative trap stiffness with a known initial diameter for *R*_*outer*_ (*D* ≈ 270 nm, Fig. 3c inset). Previous studies on protein sizes^4^,^31^,^33^ account for changes in thickness resulting from specific antibody binding to be 5.8 nm for IgG (*M*_IgG_ = 160.5 kDa) and 10.6 nm for IgM (*M*_IgM_ = 970 kDa). In our affinity assay, a measured radius increase was 7.5 ± 6.5 nm and 14.4 ± 5.6 nm for solutions of mouse IgG and mouse IgM respectively (See Supplementary Information for more details about uncertainty calculation). Relatively large uncertainty with respect to the measured increase is mainly attributable to the variations of time-averaged power. The variation is partly because of the power instability of the light source, and also partly because of the uncertainty of ~±2–3% of a photodetector equipped with a power meter (see the Supplementary Information for more details). Moreover, it is largely because of environment noise that mainly affects the light coupling from a lensed optical fiber with a micrometer-diameter tip to a nanoscale input waveguide with a cross-section of width 600 nm × height 250 nm. Note that due to a greater fractional change in polarizability for the same bound layer thickness, the sensitivity of the assay improves for smaller trapped particles (see Supplemental Figure S3).

Fig. 4d shows the stoichiometry of each binding event in consideration of the density of a bound biomolecule in a binding layer as well as the mass, for example *m*_IgG_ = 160.5 kDa ≅ 0.27 attogram (See SI for how the stoichiometry is determined in more detail). The manufacturer-quoted binding capacity of coated IgG to polystyrene particles (Spherotech, Inc) is ≈117 IgG (≈31.3 *ag*) per particle. The manufacturer-quoted binding capacity of mouse IgG (FITC-labeled) to coated anti-mouse IgG is ≈108 IgGs (≈28.7 *ag*) per particle. In comparison, the binding capacity measured in our affinity assay for mouse IgG was 124 ± 113 IgG (33.0 ± 30.0 *ag*) per particle, whereas for mouse IgM it was 57 ± 24 IgM (91.8 ± 38.5 *ag*) per particle. Despite the large uncertainties, our results of the binding capacity indicate a 1:1 binding ratio, consistent with the manufacturer-quoted binding capacity. We attribute the 1:1 binding ratio to a limited number of antibodies coated on the particle surface that are binding with abundant interacting antibodies. Although a single IgG is bivalent resulting from two light chains of binding sites, the sparsely coated antibody may have a single binding partner because of physical constraint by the IgG bound ahead (See SI and Figure S4).

Our results for the protein coated polystyrene bead experiment indicate a slightly larger bound layer thickness than reported in the previous literature. In addition to the size variation of the protein-coated polystyrene particles, our use of a Rayleigh model for a particle not strictly in the Rayleigh regime may have contributed to this deviation. The near-field optical trap is induced in a resonant optical waveguide of 600 nm width and 250 nm thickness. As shown in Fig. 2b-ii, the strong gradient field is confined in hot spots that extends in x over 300 nm greater than 270 nm diameter of a PS sphere in the width, being weakened to edges. The evanescent field penetrates in approximately 160 nm in z^23^,^34^ where the field strength decays to 1/e. In a field further than the penetration depth, a trapped particle is polarized by the field relatively weakly but the polarization effect still attributes. On the other hand, 100 nm diameter Influenza virus is within the hot spot as well as in the penetration depth where an entire viral particle is strongly polarized. Additionally, we note that theoretical estimates of protein size are based on the unhydrated mass of protein whereas our estimates account for water molecules bound to the antibody (see Figure S4). Specificity is demonstrated by the fact that we measure no change radius within experimental uncertainties (−1.7 ± 6.6 nm and −2.2 ± 5.7 nm) in the solution of a non-specific antibody (goat anti-rabbit IgG) and a buffer respectively.

**Figure 5:**
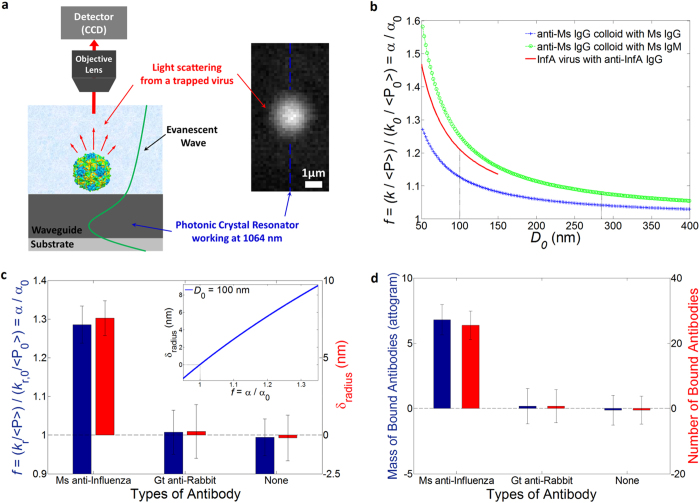
Binding of antibodies to a human influenza A H1N1 virus (*D*_*mean*_ ≈ 100 nm). (**a**) Experimental setup of the light scattering imaging. The inset shows a trapped virus particle (*D*_*virus*_ ≈ 100 nm). (**b**) Analytical plot of predicted relative power-normalized trap stiffness Note that variables including *f* and *k*_*r*_ are defined same as explained in a main text. (**c**) Measured relative power-normalized trap stiffness and radius increases for different solutions of mouse anti-influenza IgG (*N* = 3) and goat anti-rabbit IgG (*N* = 3), and a buffer (*N* = 3). *N* represents a number of independently performed experiments. The inset shows an analytical plot of the radius change to the relative polarizability for an influenza virus with *D*_0_ = 100 nm. (**d**) Stoichiometry of the antibodies to the influenza virus. All error bars are determined by (∑*σ*_*f*_^2^)^1/2^/*N* (see SI for details on *σ*_*f*_).

Next we demonstrate detection of the specific antibody to a human influenza A virus using the near-field light scattering technique (Fig. 5a). A virus particle in the evanescent field of our resonant waveguide scatters the evanescent light that allows us to image the particle with a 40x objective in the far-field. This technique allows us to develop our detection method for pathogen identification without needing to label either the virus or the antibody. In this assay we demonstrate that our method can accurately measure affinity and stoichiometry of an anti-influenza antibody to the influenza virus. Moreover we show that the sensitivity of the binding detection can be improved by trapping a smaller particle like an influenza virus (*D*_mean_ ≈ 100 nm, *σ* ≈ 10 nm) than a 270-nm diameter IgG coated colloid (Fig. 5b) for a target molecule such as IgG.

Changes in power-normalized trap stiffness are compared for different solutions of mouse anti-influenza IgG, goat anti-rabbit IgG, and a buffer (Fig. 5c). The specific binding affinity is confirmed by a radius increase of 7.6 ± 1.1 nm in the solution of mouse anti-influenza IgG. We attribute the radius increase to the specific binding of anti-influenza IgG. Specificity is demonstrated by a negligible radius increase of 0.2 ± 1.7 nm and −0.2 ± 1.5 nm in the solution of a non-specific antibody (goat anti-rabbit IgG) and a buffer respectively. Compared to the previous assay, the sensitivity was improved. We attribute this to the smaller size of viruses leading to a larger fractional polarizability change for a given bound layer, and to the better signal-to-noise ratio of the light scattering imaging technique as compared to fluorescence. The fractional uncertainty is similar in both experiments and approximately ± 0.05 (please note the error bar for Ms anti-Inf in Fig. 5c, compared with that of Ms IgG in Fig. 4c). (Also see Supplementary Information for more details). If the multiple viruses were to aggregate during an experiment, the measured trap stiffness change would not correspond to the single virus binding affinity. To prevent virus aggregation, we briefly process by vortex mixing in a dilution step, followed by sonication for a short time so that viruses are not denatured. Furthermore, because an aggregate of trapped viruses is non-spherical, the light scattering pattern can be used to distinguish a virus aggregate from a single virus particle. In addition, local heat generation in the photonic crystal resonator was considered in that generated heat could affect structures of virus and protein as well as binding kinetics. It could also contribute to uncertainty (see Supplementary Information). Based on previous investigation^23^, we estimate that the temperature increase is approximately 0.4 K, having a negligible effect on our results.

Figure 5d shows the stoichiometry of the binding antibodies using the density and mass of bound biomolecule (see SI for more details). The binding capacity of anti-influenza IgG to the virus is 6.8 ± 1.1 *ag* (26 ± 4 IgG) per virus. In comparison, specificity is shown by the negligible binding of 0.2 ± 1.4 *ag* (1 ± 5 IgG) and −0.1 ± 1.1 *ag* (−1 ± 4 IgG) per virus in the solution of goat anti-rabbit IgG and a buffer respectively. Recently, Otterstrom *et al*.^35^ reported the measured stoichiometry by counting the number of neutralizing IgG antibodies binding to a single influenza virus with two dissimilar types including H1N1 and H3N2. They used fluorescent intensity of the labeled antibody and a virus particle to measure the number based on the assumption of fractional occupancy similar to our method. Our stoichiometric results of 26 ± 4 IgG to a single H1N1 influenza virus is consistent with their results of 15 (3–37 in 95% confidence intervals) IgG. Most importantly, the comparison is possible for the same type of influenza virus H1N1 at a single particle level in similar experiment conditions; 1) the same concentration of IgG molecule (see the Sample Preparation in the Methods for the concentration in our experiments) 2) comparable fractional occupancy of our measured occupancy 0.02 (see the SI for more details about the calculation), compared with their fractional occupancy 0.09 (0.02–0.22 in 95% confidence intervals) interpolated for the same concentration.

It is noted that the stoichiometric results can vary depending upon IgG concentration as well as fractional occupancy that is experimentally determined by binding efficiency. Although the virus is covered with many hemagglutinin molecules on a viral envelope, not all of the binding sites are filled due to space constraints as the hydrodynamic radius of the IgG antibody is not negligible^33^,^36^ (*R*_*h, unhydrated*_ ≈ 5–6 nm). The IgG also have a slight negative charge, so electrostatic interactions also limit how closely they can be packed together. Thus we conclude that the number of bound anti-influenza antibodies we measure is reasonable, accounting for 1–1.5 times larger size of *R*_*h*,IgG_ to consider hydrated biomolecules. However we note that there are no preferred binding sites for sensing because the spherically shaped trapped particle is free to rotate. While many recently developed techniques^4^,^5^,^6^,^7^,^10^,^11^,^12^ are capable of virus detection, our technique enables quantitative measurements of the binding capacity of an anti-influenza antibody to a single virus.

## Conclusions

In summary, we have demonstrated a method to directly detect the binding of unrestricted biomolecules using near-field optical trapping. We have also shown the capability to measure the affinity and stoichiometry of biomolecular interactions at the attogram scale. Our measurements of the affinity and the stoichiometry of the specific antibody to the colloid were in good agreement with the manufacturer-quoted binding capacity. Our detection method does not require labeling or immobilizing either of the interacting biomolecules. We report the stoichiometric measurements for a single influenza virus and an anti-influenza antibody, which was found to be 26 ± 4 (6.8 ± 1.1 attogram) of anti-influenza antibodies per virus. Furthermore the stoichiometric results were consistent with recently published data presented by Otterstrom *et al*.^35^ that utilized fluorescent intensity to measure stoichiometry of neutralizing antibody at a single virus particle level by using TIRF (Total Internal Reflection Fluorescence). In comparison, our imaging technique of evanescent wave light scattering imaging allows a label-free method for quantifying biomolecular interactions. Moreover, high spatial resolution is achieved to localize the position fluctuations of a particle within the optical trap by fitting the scattering profile to Gaussian (see Figure S6). This allows us to attain spatial resolution of a few nanometers for tracking the Brownian fluctuations. Furthermore, our model for estimating the resulting size increase gives information with an uncertainty of ±1–2 nm for virus particles which are well within the Rayleigh regime. Our method can be utilized for studying the potential pathogenicity and virulence of rapidly mutating influenza viruses in addition to identification. Furthermore, our light-scattering detection method can be used to monitor biomolecular interactions in real time, giving new information on the kinetics of the interaction at a single molecule level as can be pursued for future work. It is noted that our method requires several thousand frames for accurate quantification which took 30 seconds to 1 minute per acquisition with our camera and imaging setup. Because a very high optical intensity is available at the center cavity of the photonic crystal resonator, we are able to observe scattered light signals from sub-100 nm particles. This technique has many potential applications in drug discovery for screening and developing drug compounds.

Our future work may include development of a new model to deal with a broad class of viruses having different shapes as well as a wide range of non-spherical particles and biomolecules^37^ using our detection methodology. Our model treats the virus particles as spheres, which was reasonable for the influenza that we studied in this work which forms a sphere in aqueous solution. To study a rod-shaped virus, such as the Tobacco Mosiac Virus (TMV), the existing model can be applied with an effective sphere approximation or a new model can be developed for the rotational trap stiffness, κ. Previously we demonstrated that our nanophotonic tweezer was able to trap a rod-shaped microtubule, constraining the rotational Brownian motion within the trap^38^.

In addition, although the current model has been developed for Rayleigh particles the particle diameter 2*r* ≤ λ/π *n*_*m*_ ≅ 245 nm, where *n*_*m*_ is refractive index of medium, *r* is radius of a trapped object, and λ is wavelength of light), we might be able to use our technique to investigate larger viruses, for example Mimivirus (*D* = 400–800 nm) and Pandoraviruses, (*D* ≈ 1 μm) as well as bacteria (*L* = 0.5–5 μm). These particles are in the Mie regime (2*r* > λ/π *n*_*m*_ ≅ 245 nm ) where calculating the response of the particle to the applied field requires a full solution of Maxwell’s equations. This could be achieved computationally, for example by Finite Difference Time Domain (FDTD) calculations^39^. In this regard, the range of suitable sizes goes from tens of nanometers up to several micrometers considering high sensitivity and a stable trap.

## Methods

### Sample preparation

Colloids and antibodies were diluted in a buffer solution of 1x phosphate buffered saline (PBS) containing 0.05% bovine serum albumin (BSA), and 0.05% Tween 20. For anti-influenza antibody 5–10 μl stock solution (antibody 2–5 μg per 1 μl stock solution) was diluted in 1 ml of the buffer solution (≅63–312 nM of IgG), and concentration of all other antibodies in a diluted solution was 1 μg/ml, which is the typical limit of detection for numerous types of biosensors^18^. Goat anti-mouse IgG coated fluorescent polystyrene particles (FITC, MFP-0252-5, *D*_mean_ = 277.3 nm, *σ* = 36.2 nm) were purchased from Spherotech Inc. (Lake Forest, Illinois). Note that the fluorescent particles are prepared by polymerizing a FITC-compatible fluorophore in the polystyrene core of particles. Mouse IgG (MG300) and mouse IgM (MGM00) antibodies were from Invitrogen Corp. (Camarillo, CA). Goat anti-rabbit IgG (A10533) antibody was from Life Technologies (Carlsbad, CA). The molecular weight of IgG antibody varies from 150 kDa to 170 kDa, depending upon types and animals. The value of 160.5 kDa for IgG antibodies in this paper was cited from the manufacturer’s information.

Swine-origin Human influenza A California/4/ 2009 (H1N1) virus (purified and UV-inactivated) was from Advanced Biotechnologies Inc. (Columbia, MD). Mouse anti-influenza A H1N1 monoclonal IgG antibody (MAB8256) was purchased from EMD Millipore Corp (Temecular, CA). Other chemicals such as Superblock blocking buffer (37580), PBS (10x concentrate, P5493), bovine serum albumin (A9647), and Tween 20 (P7949), were purchased from Sigma-Aldrich.

### Imaging and data analysis

Image acquisition was performed by a Hamamatsu ORCA-ER CCD camera controlled by Hamamatsu HCImage software. A 40x objective (LUCPlanFL N, 0.60, ∞/0–2/FN22, UIS2) was used for both the fluorescence imaging and the near-field light scattering imaging. Fluorescent particles were imaged with a FITC filter cube (excitation/emission wavelengths = 467–498 nm/513–556 nm). Fluorescence imaging was optimized with an 842 nm blocking edge BrightLine short-pass filter for exposure time of 10 ms. The manufacturer quotes a quantum efficiency of 0.45% at 1064 nm (Hamamatsu Photonics). The light scattering imaging was performed with a 641/75 nm BrightLine single-band bandpass filter (FF01-641/75-25, Transmission at 1064 nm 2.6%) for an exposure time of 20–100 μs, or a 628/40 nm BrightLine single-band bandpass filter (FF02-628/40-25, Transmission at 1064 nm 0.2%) for exposure time of 0.7–2 ms. These filters and exposure times were chosen to prevent saturation of the scattered light images. Time sequential images of the position fluctuations were obtained using the HCImage software, which were transformed to a movie file (.avi). Tracking analysis was performed using the Video Spot Tracker software developed by CISMM at UNC Chapel Hill. The FIONA kernel was used for the tracking by the software.

### Experimental measurements

In measurements, experimental parameters such as power, number of instantaneous positions to determine the trap stiffness, and uncertainty of a measurement were characterized to obtain a reliable measurement of the trap stiffness. The critical power to optically trap a *D* ≈ 270 nm colloid ranged from 1.5–2 mW (*P*_TE_) whereas that for *D* ≈ 100 nm influenza A virus ranged from 3.5–5 mW (*P*_TE_). Below this range the optical scattering force^30^ transported the particle in the propagation direction of the electromagnetic wave past the center cavity of the resonator, or the thermal energy of the particle was large enough to overcome the optical potential well, losing the optical trap. In addition, power above this range caused adhesion of a trapped particle on the resonator surface in which the relative trap stiffness ranged from 1.7 < *f* < 2.5 resulting from much smaller sampling area in the second measurement. Note that *f* = (*k*/ < *P* > )/(*k*_0_/ < *P*_0_ > ), where < *P* > is time-averaged power, the subscript 0 denotes the time when an initial measurement is performed, and *k* (=*k*_*r*_) is the radial trap stiffness defined as *k* = 2*k*_*B*_*T*/*r*_*rms*_^2^, where *k*_*B*_ is the Boltzmann constant, *T* is the temperature in K, and *r*_*rms*_^2^ = (1/*n*)∑(*x*^2^ + *y*^2^) is the variance of *n* instantaneous positions. In order to minimize external noise such as thermal excitation caused by long-time fluorescent excitation, the observation time is optimized to be 12–25 sec for each measurement with a 10 μs exposure time, resulting in 0.6 < *f* < 0.9 otherwise. (see Supplementary Information for uncertainty analysis.)

### TEM imaging of a Human influenza A H1N1 virus

Transmission electron microscopy (TEM) images of a Human influenza A H1N1 virus were taken with the FEI Tecnai F20 in STEM mode in the Cornell Center for Materials Research Shared Facilities. A staining protocol was performed prior to TEM imaging. Observed size range of the viruses was consistent with the literature^14^,^21^ which is considered to be approximately 90–110 nm in diameter. Average size was estimated at approximately 100 nm. The TEM image showed that the viruses retain viral morphology and hemagglutin (HA) in the viral envelope, allowing viable affinity assays with anti-influenza antibodies.

## Additional Information

**How to cite this article**: Kang, P. *et al*. Nanophotonic detection of freely interacting molecules on a single influenza virus. *Sci. Rep*. **5**, 12087; doi: 10.1038/srep12087 (2015).

## Supplementary Material

Supplementary Information

Supplementary Movie S1

Supplementary Movie S2

## Figures and Tables

**Figure 1:**
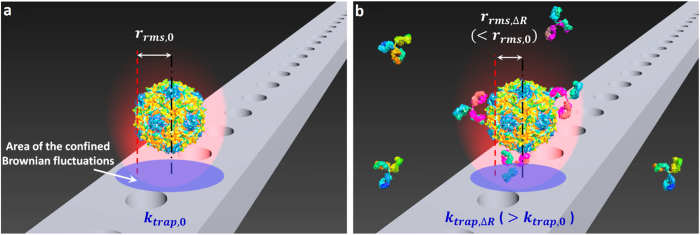
Detection principle for label-free detection of binding interactions between an influenza virus and antibodies. (i) A single influenza virus is captured in a nanophotonic near-field trap (ii) Detection of binding of an anti-influenza antibody to the virus within the optical trap. The analysis of the Brownian fluctuations determines the decreased *r*_*rms*_ (or increased *k*_*trap*_) resulting from the bindings. Note that subscript 0 denotes an initial measurement, and subscript ∆*R* denotes the measurement at equilibrium. Note that this illustration of the technique was constructed using models from the Protein Data Bank (PDB) Embedded Python Molecular Viewer (ePMV) open-source plugin, and the hemagglutinin envelope of the influenza virus used in this study was not illustrated for simplification.
