# Supplementary material for: Nanophotonic detection of freely interacting molecules on a single influenza virus
Source: Sci Rep. 2015 Jul 10;5:12087. doi: 10.1038/srep12087 (PMC4498194; doi:10.1038/srep12087)
Supplement: Supplementary Information [file srep12087-s1.doc]

Supporting Information

Nanophotonic detection of freely interacting molecules on a single influenza virus

Pilgyu Kang^1^, Perry Schein^1^, Xavier Serey^2^, Dakota O'Dell^2^, and David Erickson^1*^

^1^Sibley School of Mechanical and Aerospace Engineering, Cornell University, Ithaca, New York 14853, USA

^2^School of Applied and Engineering Physics, Cornell University, Ithaca, New York 14853, USA *Corresponding Author: de54@cornell.edu

# Design and fabrication of a photonic crystal resonator

The photonic crystal resonator design was performed using three dimensional Finite Difference Time Domain (FDTD) calculations performed with the FDTD Solutions (Lumerical Solutions Inc.) software package. NEB-31 electron beam photoresist was spun on a wafer on which a 250 nm-think silicon nitride layer was deposited on top of a 3.5 µm thermal oxide layer by low-pressure chemical vapor deposition (LPCVD, MRL Industries, Inc., a furnace for silicon nitride deposition on silicon based substrates). To reduce a charging effect during exposure, a 5-10 nm thin gold film was deposited on the resist with a thermal evaporator (CHA Industries, Inc.). It was patterned using a JEOL 9500 electron beam lithography system, followed by RIE etching (Oxford 100) using a standard nitride etch (CHF3/O2) recipe. A 3 μm-thick oxide layer to protect the device was deposited on the entire device by a sputter tool (CVC 601 sputter deposition system), followed by patterning by the lift-off process (lift-off resist, LOR-30A) to have photonic crystal resonators exposed. The fabricated device was diced in size 1 x 5 cm (KS7100 dicing saw) after spinning a 5.9 μm-thick protection layer of a photoresist (SPR 220-4.5) on a wafer. The input and output sides of a diced device were polished with Allied Multiprep polishers while sequentially decreasing size of diamond polishing papers (6 μm, 3 μm, 1 μm, and 0.5 μm) to improve coupling effiency.

# Experimental Setup

Supplementary Figure S1 shows a schematic of our experimental setup. A 1064 nm fiber coupled diode laser (LU1064M400, Lumics, El Segundo, CA) was used as a light source for an optical trapping. The laser was coupled into a silicon nitride waveguide through a lensed optical fiber. The laser diode was tuned to match the resonance wavelength of a photonic crystal resonator through temperature control with a change in center wavelength of approximately 0.3 nm per 1 K temperature rise of the laser diode[^1^](#_ENREF_1). The power coupled into the resonator was measured by focusing the light emitted from output waveguide onto a detector of a power meter. Power measurements with an optical power meter (Newport, Model 1830-C) and a photodiode sensor (Newport, Model 818-IR with a Newport 883-IR OD3 attenuator) were sampled in real time using a LabVIEW program. It is reported from a manufacturer that the photodiode sensor is calibrated with the accuracy of ±2 % at 911-1700 nm with the optical attenuator, and the power meter is specified with the measurement accuracy < ±0.2 %. A polarizer passing only TE-polarized light was placed between the focusing lens and the detector.

# Preparation of microfluidic channels

The procedure of preparation is shown in Supplementary Figure S2. To make flow channels, three holes of *D* = 500 μm were cut on a glass coverslip using a CO_2_ laser (VersaLaser VLS3.50). Punched PDMS piece was bonded to the punched cover glass by oxygen plasma bonding. A 100-µm thick parafilm spacer was cut using the CO_2_ laser to have three inlet channels combining to one channel whose width is approximately 1 mm. The parafilm spacer was laid between the cover glass and the silicon nitride chip. Next the sandwiched complex was briefly heated on a 140º C hot plate to melt the parafilm spacer and bond firmly to prevent leaking during flow and while switching solutions. Tygon tubing was inserted tightly to the holes through PDMS fixtures to inject solutions into the channels using three syringe pumps (New Era Pump Systems, Inc. NE-1000, Farmingdale, NY). Before conducting an experiment, a SuperBlock blocking buffer solution (Sigma-Aldrich, 37580) with 0.05% tween 20 (Sigma-Aldrich, P7949) was filled in the channels and incubated either over 30 min at room temperature or over 12 hours at 2-8ºC to prevent non-specific binding.

# Determination of stoichiometry of an interacting antibody

The manufacturer-quoted binding capacity of 2.89 µg antibody / mg of polystyrene particles is used to determine stoichiometry of binding antibody from the measured radius change. The number of IgG per mg of particle is calculated as *N_IgG_* = *M_IgG,total_* / *m_IgG_* = 1.084×10^13^ IgG/mg, where molecular weight of IgG is 160.5 kDa (= 0.2665 *ag*, per the manufacturer’s information). The number of polymer particles per mg are calculated as *N_ps_* = *M_ps,total_* / *m_ps_* = *M_ps,total_* / (*ρ_ps_πD*^2^) = 9.241×10^10^ particles/mg, where the density of polystyrene is 1.05 g/cm^3^, and manufacturer-quoted diameter of particles is approximately 270 nm. Therefore the binding capacity of anti-mouse IgGs to a polymer particle is calculated to be 117.4 IgGs per particle. As illustrated in Figure S4, the volume fraction occupied by the bound antibodies (*N*_IgG_ = 117.4) to the volume of an IgG-coated layer (*t* = *δ_IgG_* = 5.794 nm) determines the antibody density in a binding layer. The volume of an individual antibody is determined from dehydrated mass of an antibody as *V* = *d_IgG (or IgM)_* ^3^, where *d_IgG_* = 5.79 nm and *d_IgM_* = 10.55 nm. We use the density to determine the volume of antibodies binding to a IgG-coated colloid with a radius increase resulting from the bindings. This allows us to determine the total mass of bound antibodies, and thus find the number of the bound antibodies.

# Uncertainty of a measurement of the relative power-normalized trap stiffness

The detection method we have developed is based on fluctuation-based measurements that involve external noises such as mechanical vibrations, detector noise, and thermal noise. Determination of the uncertainty caused by these factors defines the signal-to-noise ratio, providing accurate bioassay analysis. The error propagation of power-normalized trap stiffness is determined to provide error bars in Figure 4 and Figure 5. The relative power-normalized trap stiffness is *f* = (*k*_trap,∆_*_R_* /*P*_∆_*_R_*) / (*k_trap_,*_0_ / *P*_0_), where *k_trap_* = 2 *k_B_T*/*r_rms_*^2^ is optical trap stiffness by the equipartition theorem, and$P$ is time-averaged power. The propagation error of $f$ is expressed as

$$\begin{matrix} \sigma_{f}^{2}=\left(\frac{\partial f}{\partial k'}\right)^{2}\sigma_{k'}^{2}+\left(\frac{\partial f}{\partial P'}\right)^{2}\sigma_{P'}^{2}+\left(\frac{\partial f}{\partial k}\right)^{2}\sigma_{k}^{2}+\left(\frac{\partial f}{\partial P}\right)^{2}\sigma_{P}^{2} \\ =f^{2}\cdot\left[\frac{\sigma_{k'}^{2}}{k'^{2}}+\frac{\sigma_{P'}^{2}}{P'^{2}}+\frac{\sigma_{k}^{2}}{k^{2}}+\frac{\sigma_{P}^{2}}{P^{2}}\right] \end{matrix}$$

$\sigma_{k}^{2}=\left(\frac{\partial k}{\partial T}\right)^{2}\sigma_{k}^{2}+\left(\frac{\partial k}{\partial\sigma_{r}}\right)^{2}\sigma_{\sigma_{r}}^{2}=k^{2}\cdot\left[\frac{\sigma_{T}^{2}}{T^{2}}+\frac{4^{2}\sigma_{\sigma_{r}}^{2}}{\sigma_{r}^{2}}\right]=\frac{8k^{2}}{n-1}$,

$$\sigma_{\sigma_{r}}=\sigma_{r}/\sqrt{2\left(n-1\right)}=r_{rms}/\sqrt{2\left(n-1\right)}$$

, where *n* is the number of instantaneous positions, σ*_T_* ≈ 0.4 K[^1^](#_ENREF_1), lead to σ*_T_* 2/ *T* ^2^ ≈ 0, and σ*_p_* is obtained by a power measurement in experiments. As previously studied^1^ the temperature increase due to Joule heating of water at the center cavity of a silicon nitride PhC resonator is only 0.027 K/mW, giving the benefit of substantially less thermal noise as compared to ∆*k_trap_* ≈ 7% resulting from the fluctuation-based calibration measurement by conventional optical tweezers[^4^](#_ENREF_4). From measurements the uncertainty in *f* is determined from the mean of *σ_f_* for each measurement as (∑σ*_f_ ^2^*)^1/2^ / *N*, where *N* is a number of independently performed experiments. The error bar is statistically nontrivial because it indicates the signal-to-noise ratio of our detection signal *f*. The error bars of *δ_radius_* and stoichiometries are determined from that of *f.*

In our detection method, the uncertainty in position detection originates from the integration time (which is equivalent to camera exposure time). Our analysis is based on two measurements at times before and after binding. The integration time is the same at both times. However the uncertainty in the variance of the particle position is important to consider because this uncertainty makes a major contribution to that in the trap stiffness. The statistical uncertainty in the positional variance *σ_σ_*_r_ results from the number of instantaneous positions measured, or in other words the number of frames acquired by the camera. As shown in Figure S5b, the number of *n* > 500 results in less than 1% deviation from the positional variance of a large number of *n* > 600 based upon the formula[^5^](#_ENREF_5), Standard Error (σ) = *σ* / (2*N* – 2 )^1/2^. 500-900 frames were acquired in each measurement of *k_trap_* with fluorescent microscopy at a frame rate of approximately 55-59 frame/sec, allowing 12-20 seconds of observation time for each measurement. This number of frames was chosen to avoid thermal noise by heating from the mercury lamp used to excite the fluorescence. As addressed in the Methods, long fluorescent excitation leads to f < 0.9 as a result from raised temperature *T* that increases thermal energy of a trapped particle and under-estimated *f*. In comparison, measurements with the light scattering technique had no restriction on a number of acquired frames since no external light source other than the laser was used in imaging. In these experiments 1500 to 2000 frames were acquired at the same frame rate of 55-59 frame/sec for each measurement in the virus affinity assay.

# Determination of fractional occupancy of IgG molecule in the bound layer[^2^](#_ENREF_2)


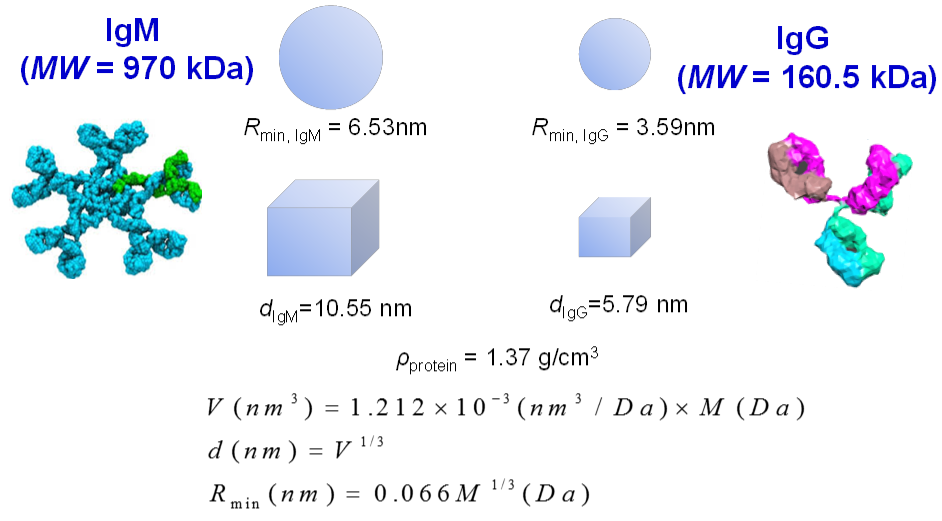


Based on experiment data, we calculate the fractional occupancy of antibody on a single particle to obtain stoichiometric results of IgG to a single influenza virus in the virus assay. We first calculate the overall surface area of a single particle for a particle of $D\cong270nm$, as

Surface area/particle = $4\pi r^{2}=\pi D^{2}=0.23\mu m=2.3\times10^{5}nm^{2}$

Next we calculate occupying area of a molecule by assuming a packed cube from molecule volume of hydrodynamic radius, *R*_min_ that is determined from molecular weight (MW), *M* in Dalton (*Da*).

$d_{IgG}=5.8nm$ and $d_{IgM}=10.6nm$

This allows us to determine occupied area by IgG/IgM molecule as

$A_{IgG}=33.6nm^{2}$, $A_{IgM}=111.4nm^{2}$

Then , the fraction of area occupied by coated IgG per paritcle can determine fractional occupying area by overall bound molecules that we know the number of bound molecules.

$$f_{area}=\frac{N_{IgG}\times A_{IgG}}{A_{particle}}=\frac{117\times111.4nm^{2}}{2.29\times10^{5}nm^{2}}=0.057=5.7\%$$

Finally we determine the fractional volume occupancy (FVO) that represents fractional volume occupied by bound IgG per particle

$$FVO=f_{vol,IgG}=\frac{N_{IgG}V_{IgG}}{V_{\text{coated IgG layer}}}=\frac{117\times194.1nm^{3}}{1.27\times10^{6}nm^{3}}=0.018=1.8\%$$

$$f_{vol,water}=1-f_{vol,IgG}=0.982=98.2\%$$

Note that the entire volume of bound IgG layer *V*_coated IgG layer_ in the above is calculated as

$$\begin{matrix} V_{\text{coated IgG layer}}=\frac{\pi}{6}\times\left[D_{IgG-ps}^{3}-\left(D_{IgG-ps}-2\times d_{IgG}\right)^{3}\right] \\ =\frac{\pi}{6}\times\left[270^{3}-\left(270nm-2\times5.8nm\right)^{3}\right]=1.27\times10^{6}nm^{3} \end{matrix}$$

In this aspect, the bound layer is illustrated in Figure S4 that accounts for the fractional occupancy.

# Supplementary Figure S1


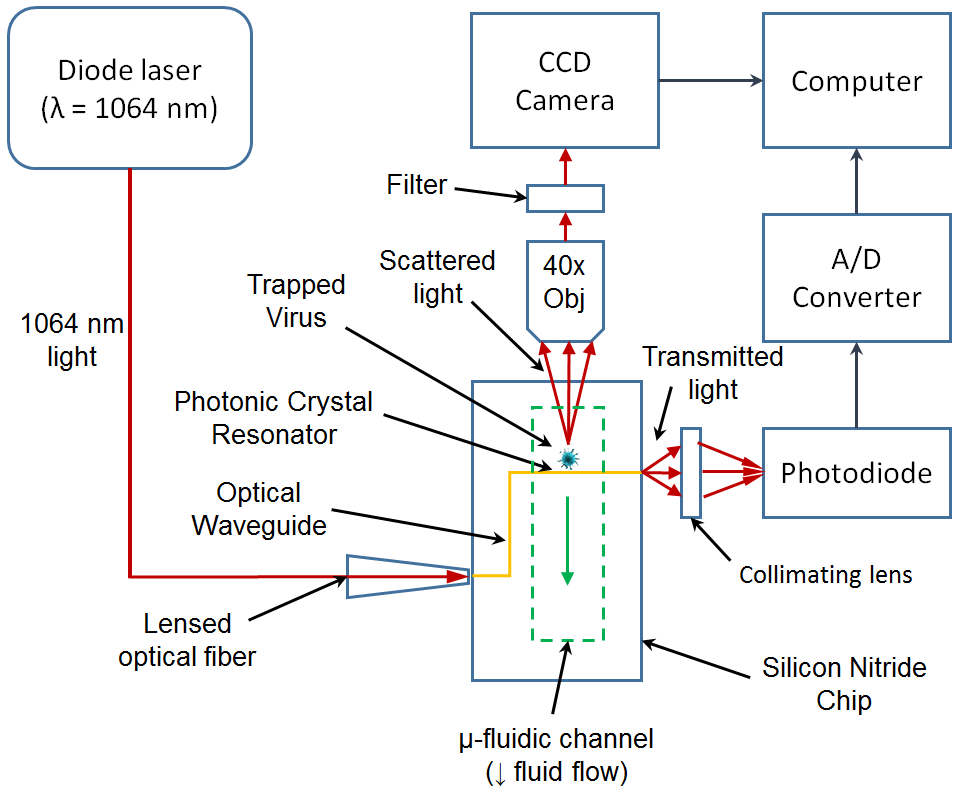


Figure S1. Schematic of the experiment setup. Imaging conditions and types of filters are described for a protein coated fluorescent particle and an influenza virus respectively in the Methods.

# Supplementary Figure S2


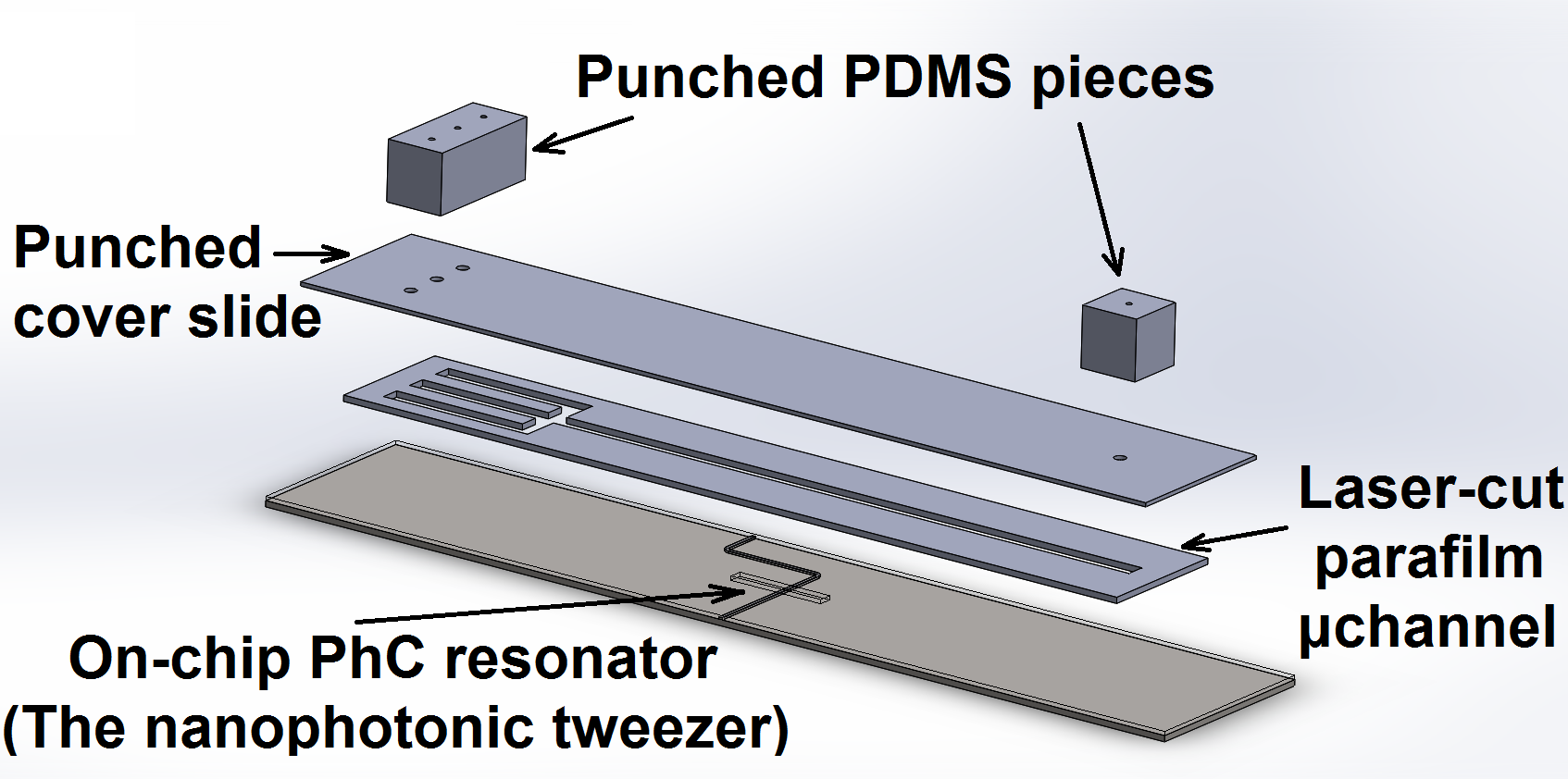


Figure S2. Schematics of preparation of a microfluidic channel. The preparation of assembly components and fabrication of a photonic crystal resonator device are described in the supplementary information above.

# Supplementary Figure S3


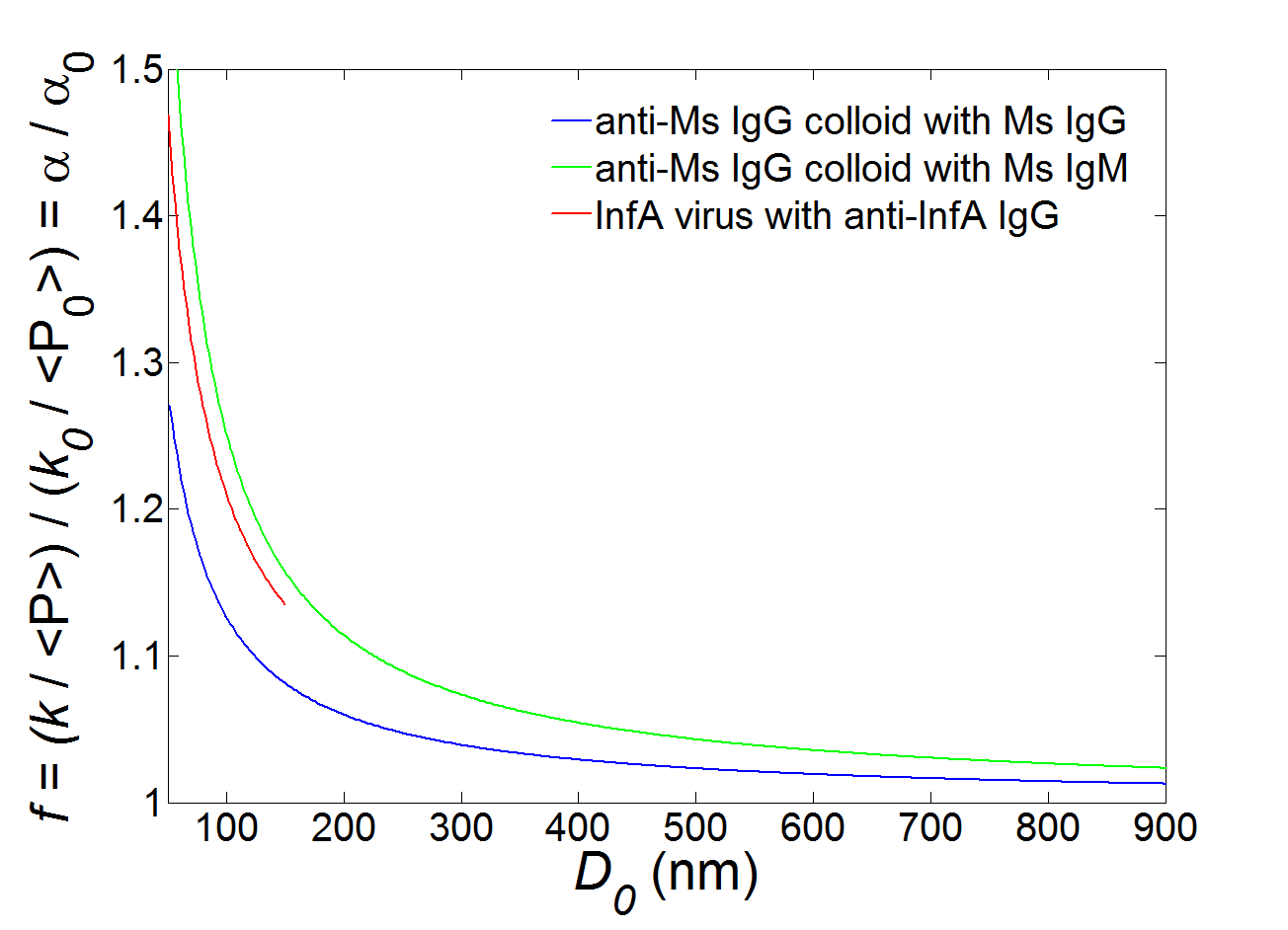


Figure S3. Sensitivity of the detection of bound antibody to different sizes. This analytical plot is calculated from Eq. 2 for varying sizes of *R_inner_* and the sizes of antibodies such as IgG and IgM. Following indices *n_PS_* = 1.59 for a polystyrene (PS) particle, *n_IgG_* = 1.41 for an antibody, and *n_virus_* = 1.48 for an influenza virus[^7^](#_ENREF_7) were used for this plot. A blue solid line shows the sensitivity of the detection of bound mouse IgG to a goat anti-mouse IgG coated polystyrene particle whose size ranges in 50 – 900 nm. A green solid line indicates the sensitivity of the detection of bound mouse IgM to the anti-mouse IgG coated colloid whose size ranges in 50 – 900 nm. A red solid line indicates the sensitivity of the detection of bound anti-influenza IgG to a human Influenza A H1N1 virus whose size ranges in 50 – 150 nm. Thickness of bound antibody layers is considered to be *∆R_IgG_* = 5.79 nm (*M_IgG_* = 160.5 kDa) and  *∆R_IgM_* = 10.55 nm *M_IgM_* = 970 kDa). This graph demonstrates that the sensitivity for detecting the binding of a certain size of a biomolecule increases if the partner molecule previously trapped is smaller. This plot shows that the sensitivity of the virus assay to detect anti-influenza IgG was higher than that of the characterization assay with the colloid.

# Supplementary Figure S4


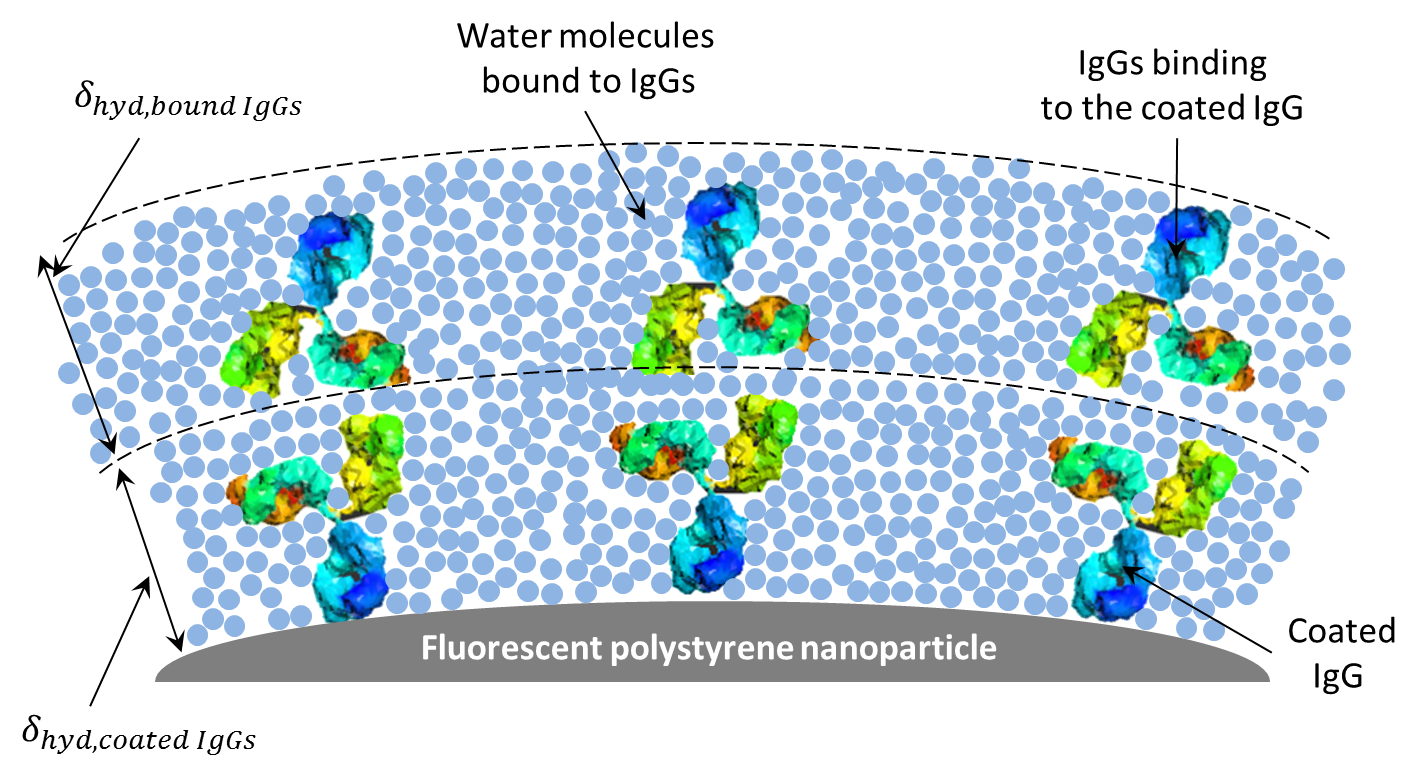


Figure S4. Illustration of an IgG coated fluorescent nanoparticle and binding IgGs. Note that the IgG coated layer and bound IgG layer retain water molecules[^6^](#_ENREF_6). This must be accounted for when calculating the number of bound biological molecules per unit volume. The colloidal particle used in these validation experiments is on the same order of magnitude in size (hundreds of nm) as the virus particles studied, and the same type of biomolecule, IgG is used in both experiments.

# Supplementary Figure S5


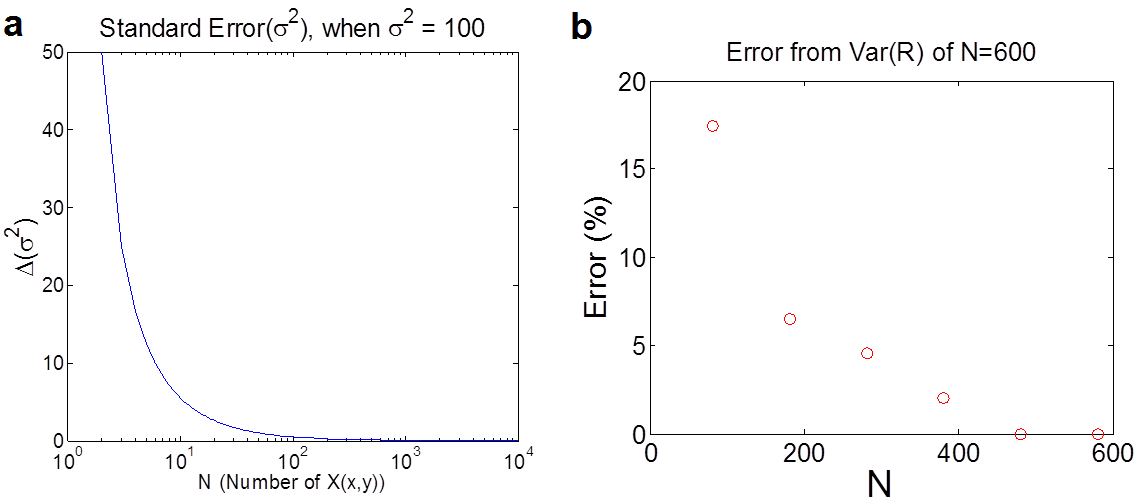


Figure S5. Uncertainty of the standard deviation of the set of *N* frames. (a) Theoretical error estimation of positional variance (*σ* ^2^) that is equal to *r_rms_*^2^ as a function of the number of frames that determines the trap stiffness as *k_trap_* = 2*k_B_T*/*r_rms_*^2^, where *k_B_* is the Boltzmann constant, *T* is the temperature in K, and *r_rms_*^2^ =(1/*N*)∑(*x*^2^ + *y*^2^) is the variance of *N* instantaneous positions. The figure S1 (a) shows a deviation of the variance as a function of the number of frames *N* with respect to *σ* ^2^= 100 (*N* = 10^4^), based upon the formula of uncertainty of standard deviation of the set of *N* measurements, *σ* / (2*N* – 2 )^1/2^. Note the uncertainty decreases as the number of frames used increases. As the number of frames increases over 10^2^, the uncertainty becomes negligible. (b) Deviation of positional variance in percentage as a function of the number of instantaneous positions with respect to reasonably large number of the instantaneous positions (*N* =600) with an experiment result of a *D* = 270 nm IgG coated fluorescent particle in a buffer solution (a calibration experiment for the case of no bindings to the trapped particle).

# Supplementary Figure S6


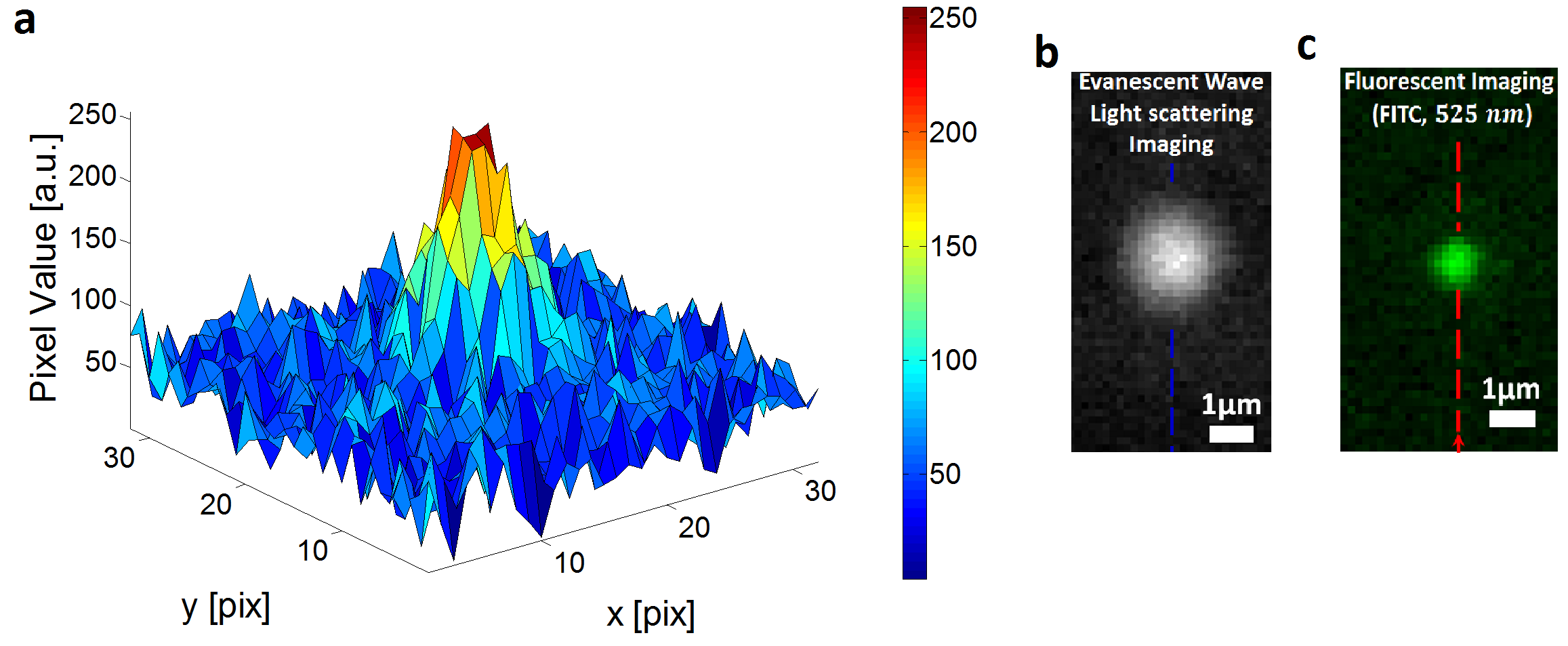


Figure S6. Intensity profile and comparison of images obtained by different imaging techniques (a) Images obtained by evanescent wave light scattering imaging and conventional fluorescent imaging is represented as pixel value in each frame where the centroid is fitted by the least-squares of 2D Gaussian to the intensity profile. Tracking of the centroids allows the localization of particle positions with a few nanometer accuracy so as to quantify the Brownian fluctuations with high spatial resolution, surpassing optical resolution limit. (b) An optically trapped influenza virus (*D* ≈ 100 nm) imaged with evanescent wave light scattering by a 40x objective lens (c) An optically trapped IgG-coated fluorescent polystyrene particle (*D* ≈ 270 nm). (See the Methods for more details about imaging condition and image processing)

# Supplementary Movies

## Supplementary_Movie_S1.avi

Bioaffinity assay with a *D* = 270 nm goat anti-mouse IgG coated fluorescent polystyrene particle by fluorescence microscopy. The particle is optically trapped on a silicon nitride photonic crystal resonator. The solution is switched to mouse IgG and allowed to incubate for 30 minutes to saturate binding to the trapped polymer particle. The antigen-antibody-particle complex is released by turning off the laser.

## Supplementary_Movie_S2.avi

Affinity assay with an influenza A virus using the evanescent wave light scattering technique. The virus is optically trapped on a silicon nitride photonic crystal resonator. The solution is switched to mouse anti-influenza IgG and allowed to incubate for 30 minutes to saturate binding to the trapped viral particle. Light scattering after releasing the antibody-virus complex by turning off the laser is observed with the laser on.

# References

1 Chen, Y. F., Serey, X., Sarkar, R., Chen, P. & Erickson, D. Controlled Photonic Manipulation of Proteins and Other Nanomaterials. *Nano Lett* **12**, 1633-1637 (2012).

2 Erickson, H. P. Size and Shape of Protein Molecules at the Nanometer Level Determined by Sedimentation, Gel Filtration, and Electron Microscopy. *Biol Proced Online* **11**, 32-51 (2009).

3 Yu, C. & Irudayaraj, J. Quantitative evaluation of sensitivity and selectivity of multiplex nanoSPR biosensor assays. *Biophys J* **93**, 3684-3692 (2007).

4 Florin, E. L., Pralle, A., Stelzer, E. H. K. & Horber, J. K. H. Photonic force microscope calibration by thermal noise analysis. *Appl Phys a-Mater* **66**, S75-S78 (1998).

5 Ahn, S. & Fessler, J. A. Standard errors of mean, variance, and standard deviation estimators. (2003).

6 Voros, J. The density and refractive index of adsorbing protein layers. *Biophys J* **87**, 553-561 (2004).

7 Wang, S. P. *et al.* Label-free imaging, detection, and mass measurement of single viruses by surface plasmon resonance. *P Natl Acad Sci USA* **107**, 16028-16032 (2010).

8 Gu, H. W., Xu, K. M., Xu, C. J. & Xu, B. Biofunctional magnetic nanoparticles for protein separation and pathogen detection. *Chem Commun*, 941-949 (2006).

9 Murphy, R. M. *et al.* Size and Structure of Antigen-Antibody Complexes - Electron-Microscopy and Light-Scattering Studies. *Biophys J* **54**, 45-56 (1988).
